# Receptor Binding Domain Based HIV Vaccines

**DOI:** 10.1155/2015/594109

**Published:** 2015-01-15

**Authors:** Huan Liu, Wenwen Bi, Qian Wang, Lu Lu, Shibo Jiang

**Affiliations:** ^1^State Key Laboratory of Virology, Wuhan Institute of Virology, Chinese Academy of Sciences, Xiaohongshan 44, Wuhan 430071, China; ^2^Key Lab of Medical Molecular Virology of MOE/MOH, Shanghai Medical College, Fudan University, 130 Dong An Road, Xuhui District, Shanghai 200032, China; ^3^Lindsley F. Kimball Research Institute, New York Blood Center, New York, NY 10065, USA

## Abstract

This paper analyzes the main trend of the development of acquired immunodeficiency syndrome (AIDS) vaccines in recent years. Designing an HIV-1 vaccine that provides robust protection from HIV-1 infection remains a challenge despite many years of effort. Therefore, we describe the receptor binding domain of gp120 as a target for developing AIDS vaccines. And we recommend some measures that could induce efficiently and produce cross-reactive neutralizing antibodies with high binding affinity. Those measures may offer a new way of the research and development of the potent and broad AIDS vaccines.

## 1. Introduction

The global pandemic of acquired immunodeficiency syndrome (AIDS) caused by HIV continues to expand, and this is still a serious global health problem. It is reported that about 34 million people are living with HIV globally in 2012. The Joint United Nations Programmed on HIV and AIDS (UNAIDS) reported that 2.5 million people became newly infected in 2011 and 1.7 million people died that year from AIDS related causes [1]. Unfortunately, there is no effective preventive vaccine in the world. Therefore, whether we can get safe and effective preventive vaccines is a hotspot and difficulty in the field of current international AIDS research.

So far, AIDS vaccine development has followed three major trends. The first wave of candidate vaccines was designed of envelope proteins and synthetic peptides mimicking gp120 epitomes, mainly aimed at inducing neutralizing antibodies (Nabs) [2]. However, induction of potent and broadly cross-reactive neutralizing antibody responses remains a major challenge confronting the development of HIV vaccines because of the high diversity of gp120. The high glycosylation, large conformational changes, and steric restriction of the epitopes in gp120 during receptor binding and membrane fusion processes prevent antibodies from accessing the potentially vulnerable sites [3]. AIDSVAX, VaxGen's gp120-based AIDS vaccine, failed in clinical trials, raising questions about the strategy of using viral Env protein to induce neutralizing antibody responses.

The second wave of candidate vaccines was designed of vectors, such as weakened adenovirus that encodes the HIV-1 proteins Gag, Pol, and Nef, to stimulate HIV-1-specific cellular immunity. One such vaccine candidate is V520 developed by the researchers at Merck & Co. [4–6]. Although this vaccine could elicit strong immune response and showed protection in animal models, the clinical trial for V520 (STEP) was discontinued in 2007 because this vaccine did not provide protection in vaccinated human subjects and was even associated with increased risk of HIV infection in some recipients [7–9].

The third wave of candidate vaccines was aimed at induction of both humeral and cell-mediated immune responses with heterogonous priming-booster strategies [3, 10]. One representative vaccine is the combination of AIDSVAX, as noted above, and Sanofi Pasteur's vector-based vaccine, ALVAC. The results from the phase III clinical trial (RV144) for ALVAC/AIDSVAX showed modest efficacy (31.2% reduction of HIV infection rates compared with those in the placebo group) [11]. Further analysis of the clinical samples revealed that induction of antibodies against gp120 by the vaccine may contribute to the protection of the participants from HIV-1 infection. For example, some study in order to identify the risk of HIV-1 infection in RV144, two sequential sets of analyses of plasma specimens shown that the levels of IgG antibodies (Abs) specific for gp120 V2 were correlated with decreased the risk of infection, the level of IgA Abs reactive with envelope glycoproteins correlated with decreased vaccine efficacy [12]. And four monoclonal antibodies (CH58, CH59, HG107, and HG120) from RV144 vaccines have been described [13]. CH58 and CH59 could bind to gp120 vaccine antigen and also to a HIV-1 envelope variable region 2 peptide. Epitope mapping showed that they could recognize the residues of lysine (K) at position 169. When the vaccine envelope at residue 169 was mutated, the neutralization was reduced or abrogated. In the case of the RV144 vaccine, the variable region as the target for antibodies correlated with increased vaccine efficacy. It has been demonstrated in the past that broadly neutralizing antibodies can bind glycans and variable region (V1 and V2) residues around position 169 [14]. The crystal structures of the CH58 and CH59 showed that they recognize similar V2 residues in completely different conformations. It is suggested that the V2 regions may exist in multiple conformations. These promising results have encouraged researchers to refocus on the studies of structure, function, antigenicity, and immunogenicity of gp120 in order to identify the critical functional regions containing relatively conserved neutralizing epitopes that may induce potent and broadly cross-reactive neutralizing antibodies.

In previous studies, we have attempted to use the receptor binding site (RBD) of the virus such as MERS-CoV [15–17], SARS [18, 19], and avian influenza A virus [20, 21] models as an antigen for vaccine design. In practice, this strategy has achieved very good results: it induced high titers of neutralizing antibodies and provided the effective protection for animals attacked by virus in animal experiments. At the same time, the full-length virus envelope S protein as immunogen may cause the immune system to overreact, which leads to negative effects such as aggravation of illness; thus a shorter RBD area can effectively avoid this problem, such as SARS vaccine [22]. The same strategy may also be suitable for HIV vaccine design, especially after the finding of HIV RBD neutralizing antibody VRC01, VRC-PG04, 3BNC60, and the HJ16; the conservative area of RBD was confirmed once again to play an important role in the process of virus infection. They can induce highly effective broad spectrum neutralizing antibodies. Therefore, the conservative area of RBD is still a very important target. And, RV144 vaccines clinical trial also suggested that the levels of IgG specific for HIV-1 Env were inversely correlated with vaccine protective effect; thus there may be some areas which could increase the infection on the full-length Env of HIV-1, and shorter gp120 RBD may reduce this effect. Of course, there are some differences in RBD between HIV and MERS-CoV, SARS, and influenza, making it much harder to design of its vaccine based on this strategy. In this review, we will focus on the characteristics of HIV RBD, analyze the RBD of gp120 as a target to develop HIV vaccines, propose some new ideas and the new specific-boosting strategies based on these issues, and provide new thoughts for the research and development of an effective AIDS vaccine.

## 2. The Structure and Function of gp120 and Its Receptor Binding Domain (RBD)

**Figure 1:**
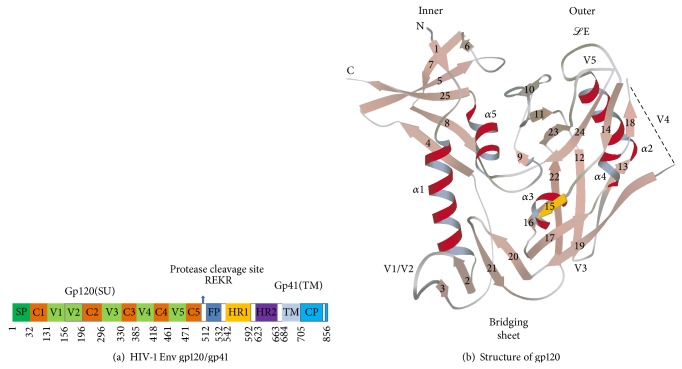
(a) Primary structure of HIV-1 Eve glycoprotein and sequence variations in different regions of the Eve. Approximate locations conserved regions (C1 to C5) and variable regions (V1 to V5) are shown along with the numbering scheme of amino acids. The cross-linking disulfide bonds connecting various segments. SP: surfactant protein; FP: fusion peptide; HR: heptad repeat; TM: transmembrane domain; CP: cytoplasm domain. (b) Crystal structures [25] of gp120. The inner domain in the left portion of core gp120, the outer domain in the right portion, and the 4-stranded sheet at the bottom left of gp120 as the bridging sheet (*β*3, *β*2, *β*21, and *β*20). *α*-Helices are depicted in red and *β*-strands in salmon, except for strand *β*15 (yellow), which makes an antiparallel *β*-sheet alignment with strand C terminal of CD4. Connections are shown in grey, except for the disordered V4 loop (dashed line) connecting *β*18 and *β*19.

Like other viral envelope glycoproteins the HIV Env consists of two subunits, the surface glycoprotein (SU), which is responsible for binding to receptor molecules, and the transmembrane glycoprotein (TM), which mediates fusion of the viral membrane with the cell membrane (Figure 1(a)). Entry of HIV into the target cell is initiated by the binding of viral envelope spike protein (Env) surface subunit gp120 to the primary receptor CD4 via its CD4 binding site (CD4bs) [23] resulting in the exposure of the coreceptor binding domain or CD4-induced (CD4i) conformation [23, 24].

Binding of CD4i to a coreceptor, CCR5 or CXCR4, causes a series of conformation changes in the Env transmembrane subunit gp41, leading to the fusion of the viral envelope with the target cell membrane [26, 27]. Therefore, gp120 plays an important role in viral binding to the receptor and coreceptor, as well as entry into the target cell. As such, the RBD in gp120 may serve as an attractive target for AIDS vaccine development.

The HIV-1 gp120 consists of five conserved regions (C1 to C5) interspaced with five variable regions (V1 to V5) [28]. Regions V1 to V4 create surface-exposed loops anchored by disulfide bonds at their base. The third variable (V3) loop of gp120 can be separated from the main body of gp120 for direct contact with coreceptors during viral fusion [28], making it the key determinant in gp120 for defining coreceptor selection, that is, CCR5 or CXCR4.

Crystallographic analyses of the structures of truncated gp120 of HIV-1 and SIV, free or in complex with sCD4 and/or neutralizing antibodies, indicate that the CD4-bound conformation of a gp120 monomer is comprised of an inner domain and an outer domain linked by a bridging sheet.  Figure 1(b) showed that the core gp120 comprises 25 *β*-strands, 5 *α*-helices, and 10 defined loop segments. The outer domain consists of a six-stranded mixed-directional *β*-sheet which clamps helices, *α*2, and a seven-stranded antiparallel *β*-barrel. A structurally conserved cavity is localized between the inner and outer domain of gp120 into which Phe43 of the CD4 on the target cell protrudes during the interaction between viral gp120 and cellular CD4 receptor [25]. Different with SARS and influenza, HIV has a stronger conformational CD4bs which locate in multiple areas of the C1–C5 in gp120. It is a discontinuous determinant consisting of the residues in the cavity, including Trp112, Val255, Thr257, Asp368, Glu370, Ile371, Trp427, Val430, and Gly473 [25], in addition to several residues outside the cavity, such as Arg59. The coreceptor binding sites are located in the gp120 bridging sheet that is formed during the structural rearrangements induced in gp120 upon CD4 binding. Furthermore, CD4-induced conformation changes of the gp120 variable domains allow the V3 loop to bind to the coreceptors, mainly with the second extracellular loop (ECL2) in CCR5 or CXCR4 [29].

## 3. The CD4 Binding Domain in gp120 as a Target for Developing AIDS Vaccines

Because of the critical role of RBD in gp120 in viral binding, CD4bs is thought to be an ideal target for eliciting broadly cross-reactive neutralizing antibodies. The first CD4bs-specific human monoclonal antibody (mAb), b12, was identified in 1994 by Burton and colleagues [30]. It can bind to gp120 via CD4bs, compete with soluble CD4 for binding to gp120 [31], and strongly neutralize a broad spectrum of T cell derived laboratory (TCLA) strains and primary HIV-1 isolates [30, 32, 33]. Most recently, several more potent CD4bs-specific neutralizing mAbs, including VRC01, VRC-PG04, 3BNC60, and HJ16, have been identified by different groups [34–38]. The mAb VRC01 neutralizes about 90% of different HIV clades tested [37, 39]. PotentVRC01-like (PVL) HIV-1 antibodies derived from the VH1-2^*^02 germline allele target the conserved CD4 binding site on gp120. Available structure information showed that Trp50 and Asn58 of VRC01-like antibodies are within hydrogen bonding distance of Asn280 and Arg456 of gp120, respectively, and Trp102 hydrogen bonds with Asn279 of gp120. Crystal structures of several such Abs bound to gp120 [38, 40, 41] revealed molecular details of their neutralization mechanisms and facilitated structure-based rational design to improve their potency and breadth. These findings, and the surprising subsequent discovery of VRC01-like antibodies in different HIV-1 seropositive human donors, aroused the enthusiasm of the people for the CD4bs as vaccine targets [36–38]. These spotlight results markedly promote interest in studying CD4bs neutralizing antibodies and designing immunogens to elicit these NAbs. Interestingly, some of the CD4bs-specific mAbs have no neutralizing activity or can only neutralize some HIV-1 TCLA strains, dependent on their binding affinity to the HIV-1 gp120/gp41 trimers. The mAb b12 has similar binding affinity to the gp120 monomer and trimer on the surface of infected cells [42], while those CD4bs-specific mAbs with no neutralizing activity have weaker affinity to the gp120/gp41 trimer. This suggests that the mAbs without strong neutralizing activity cannot recognize or access the native conformational epitopes on the gp120/gp41 [43]. Although the breadth of neutralization by b12 is somewhat limited either by variations in sequence of the CD4 binding loop or by distal mutations that seem to affect accessibility to its epitope on the native Env trimer [44]. Therefore, in designing CD4bs-based AIDS vaccines, it is essential to maintain the conformational neutralizing epitopes on native gp120/gp41 trimer. Some groups aimed to construct gp120 or gp140 trimer with native conformation by linking the extracellular region of gp41 covalently to the gp120 and then fusing it into the GCN4 trimer domain and expressing the soluble gp140 oligomer. Other groups expressed the uncleaved and unactivated gp140 using the fold on trimer domain of T4 phage pilus [45–47], or packaging native gp160 with its cytoplasmic domain deleted (gp160 ΔCT) using protein liposome [44]. Unfortunately, however, the neutralizing epitopes in most of these trimers showed very weak immunogenicity, probably because the CD4bs in these trimers is in a recessed pocket, making it hard for antibody to access. Meanwhile, the nonneutralizing epitopes in gp120 have strong immunogenicity, which observably weakens the immune system to produce antibodies against the neutralizing epitopes. Therefore, enhancing the immunogenicity of neutralizing epitopes, while suppressing the immunogenicity of nonneutralizing epitopes, is an important approach in designing CD4bs-based vaccines. For instance, the antigenicity and immunogenicity of the immunodominant nonneutralizing epitopes can be abrogated in several ways, including induction of conformational changes by mutation or glycan shield of carbohydrate molecules [48, 49]. Recently, a human CD4bs (CD4 binding site) monoclonal antibody 1F7 that separated from immortalized peripheral blood lymphocytes from blood of HIV-1 positive volunteers was reported. Although the epitope of 1F7 had not been described until the current study, it is important to note that 1F7 recently proved induced shedding of gp120 from spikes of primary HIV-1; b12, but not VRC01 also mediated the shedding effect [39, 50, 51]. Here again, 1F7 shares a functional property with b12 that VRC01 lacks, may be attributable partly for the conformation change on binding to the spikes with b12 and 1F7 but not with VRC01. 1F7 seems to have more in common with b12 about the weak neutralization broad and has no sensitivity to V5 mutations. The perspective of a different way and positioning of antibody is crucial to recognize CD4bs effectively, considering the antibody Fab arm is about twice the width of the CD4 [52]. Hence, this study showed that the positioning of the gp120 plays an important role for antibody to effective CD4bs recognition.

## 4. The Coreceptor Binding Domain in gp120 as Another Target for Developing AIDS Vaccines

Available evidence suggests that biologically important coreceptors for HIV are the chemokine receptors CXCR4 and CCR5 [53]. They consist of an extracellular N-terminus, an intracellular C-terminus, seven *α*-helical transmembrane domains with several conserved Pro residues, and three intracellular and extracellular loops composed of hydrophilic amino acids [54, 55]. These hydrophilic and charged residues interact with polar faces and hydrogen bond with the ring hydroxyl groups [56]. Thus, based on the hydrophilic amino acids design peptides which containing polar groups interact with hydrophilic amino acids. The N-terminus of CCR5, which contains several sulfated tyrosines, plays a critical role in the CD4-dependent association of gp120 with CCR5 and in viral entry. Some studied showed that Tyr10 and Tyr14 in CCR5 play a critical role in the interaction of CCR5 with V3, such as the sulfated human mAb 412d which interacts with the CCR5-binding site in V3 [57].

As mentioned above, the binding of CD4 to gp120 induces formation of the bridging sheet and exposure of the coreceptor binding sites in gp120, including the V3 loop region. Some of these conserved CD4-induced (CD4i) epitopes in the gp120 bridging sheet contain neutralizing epitopes that can elicit broadly cross-reactive neutralizing antibodies, such as 17b [58, 59]. A number of experimental findings confirm the existence of these epitopes. First, the affinity of soluble gp120 to coreceptors could be greatly enhanced after CD4 binding to gp120 [48, 58]. Second, soluble CD4 (sCD4) could markedly enhance the fusion efficiency between HIV-1 and the cells expressing a coreceptor, CCR5 or CXCR4 [60, 61]. Third, CD4 binding could enhance the affinity of human mAbs 17b and 48d binding to gp120. Several groups have mimicked the conformation of CD4i epitopes to induce NAbs. However, only the NAbs against the relatively conserved structure of V3 loop, which interacts with CCR5/CXCR4, have succeeded in exhibiting broadly cross-reactive neutralizing activity [62–68].

## 5. Quaternary Binding Sites in gp120 as Targets for Developing AIDS Vaccines

**Figure 2:**
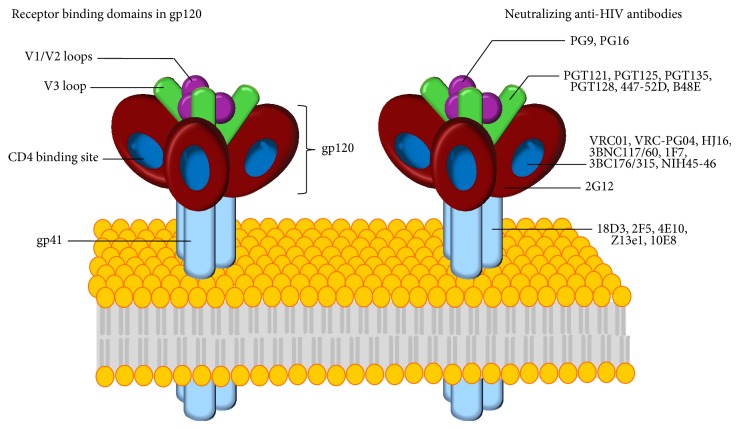
The envelope of HIV-1 carries spikes. (a) Each spike is made of three molecules of the surface glycoprotein gp120 and three molecules of the transmembrane glycoprotein gp41. Glycoprotein gp120 contains variable V1/V2 and V3 loops, as well as the binding site for CD4. (b) The binding sites of broadly acting and potent HIV-1-specific neutralizing antibodies are shown as coloured circles. The target sites investigated by the new studies (a site at the base of the V3 loop [64] and the CD4 binding site) are marked by green circles.

The quaternary sites include variable loops and glycosylation sites [69]. Some neutralizing anti-HIV-1 antibodies binding the variable loops were identified, such as PG9, PG16 [51], CH01-04 [14], and PGT14 [70] target site on V2 or V3 loops of gp120; PGT121, PGT125, PGT128, and PGT135 interact with gp120 of V3 loops (Figure 2). PG9 and PGT 128, which rely primarily on backbone interactions with V1-V2 and V3 respectively, to form main chain *β* strand association [71, 72]. A substantial proportion of the broadly neutralizing antibodies (bnAbs) identified in certain HIV-1 infected donors recognize glycan-dependent epitopes on HIV-1 gp120. Binding glycosylation site's neutralizing anti-HIV-1 antibodies like monoclonal antibody PG9 and 2G12 were determined [73] and found that PG9 and 2G12 binding to dual glycans on a scaffolded glycopeptide and high-mannose glycans, respectively [74]. A number of other glycan-dependent bnAbs include PG16, PGTs 121–123, PGTs 130-131, and PGTs 142–145. With the appearance of new single-cell cloning techniques [61, 75], the hopeful bNAbs have been greatly expanded to include the number of the quaternary specific Abs, including glycosylation and its antigen gp120 variable cycle [64, 76]. Neutralizing antibodies need to access the fragile sites in coated trimer with the AIDS virus. However, the complicated quaternary structure of the trimeric envelope spike efficiently protects functionally important domains [77]. Some researchers analyse these broadly neutralizing antibodies' crystal structures and found that they interact with gp120 by van der Waals, hydrogen bonds, and salt bridges. Therefore, we can use some method by modifying antibodies to improve the interaction with gp120 and to gain more effective neutralizing antibody. At the same time, we expect that the antibodies induced by vaccines can act through allosteric effect by binding different sites of the variable loops, implying they may play a significant role in HIV-1 disease.

It is now widely accepted that nonneutralizing antibodies (Non-Nabs) as well as neutralizing antibodies (NAbs) display inhibitory activities against HIV-1 replication in vitro based on different mechanisms [78]. In the RV144 Thai phase III trial, a reduced risk of infection in vaccinated individuals did not correlate with serum neutralizing activities but with high concentrations of anti-V1-V2 Non-Nabs [79], providing additional support for a possible contribution of Non-Nab functions to protection [80]. Thus we conclude that some nonneutralizing antibodies for V2 may also have effect to prevent HIV-1 infection.

Most recently, a number of CD4i antibodies including 17b, X5, E51, 48d, and 412d recognize highly conserved CD4i epitopes were reported that overlap to various extents with the coreceptor binding site (Table 1). The epitopes of 17b and X5 are known after the determination of the gp120 structure complexed with Fab 17b or Fab X5. These findings suggest that CD4i epitopes can also serve as targets for developing AIDS vaccines. 18D3, 2F5, 4E10, and 10E8 antibodies target NHR pocket or the transmembrane glycoprotein gp41 of HIV-1 (Figure 2), which anchors the viral envelope to the underlying virus particle [81–84].

The other antibodies 2G12 and b12 target on the outer envelope protein gp120. Some study showed that 2G12 binds to HIV-1 envelope glycans [81], whereas b12 blocks the binding site to CD4 [85], the main receptor for HIV on the surface of immune T cells. However, these coreceptor binding epitopes in gp120 on the virions during the natural infection process are unlikely to elicit immune responses based on steric hindrance and time limitation because exposure of these epitopes (from gp120-binding to CD4 to gp120-binding to a coreceptor) to the immune system is very limited. Therefore, the design of an immunogen containing the CD4i epitopes that stably maintain the native conformation is critical to the development of a coreceptor binding site-based AIDS vaccine.

## 6. Conclusion and Prospection

Our previous studies have demonstrated that the RBD in the spike protein of severe acute respiratory syndrome- (SARS-) associated coronavirus (SARS-CoV) is one of the most important targets for developing SARS vaccines. Similarly, the RBDs, including the CD4bs and CD4i, in the HIV-1 gp120 are also attractive targets for the development of AIDS vaccines because of the recent identification of several series of human broadly cross-reactive neutralizing antibodies targeting the CD4bs [34–38] and CD4i epitopes [39, 62, 70] in gp120. However, unlike the RBD of SARS-CoV spike protein that consists of a short fragment (about 200 amino acid residues), the CD4 and coreceptor binding domains in gp120 of HIV-1 are formed by a number of amino acids discontinuously spreading in almost the entire gp120 molecule. This complicates the design of AIDS vaccines containing the CD4bs and/or CD4i epitopes that maintain native conformation. Transitory exposure of the CD4i epitopes and the presence of nonneutralizing immunodominant epitopes in gp120 are additional obstacles. Therefore, to design vaccines with the ability to induce highly potent and cross-reactive neutralizing antibodies with high binding affinity, we recommend the following strategies: (1) construct an immunogen containing the discontinuous sequences of CD4bs and/or CD4i epitopes, and the natural trimeric conformation is essential; (2) induce conformational changes by mutation or glycan shield of the nonneutralizing immunodominant epitopes from the immunogen in order to reduce the immunogenicity of these epitopes; and (3) add an immune potentiation motif to the immunogen in order to enhance the immunogenicity of the RBD based vaccine.

Based on these strategies, we proposed a new strategy—specific-boosting strategy. The core of this new strategy is to inspire the specific neutralizing antibody to RBD epitopes based on the natural Env trimer by using the priming-booster mode. Using the plasmid, which can be expressed the natural trimeric Env, or protein (such as ALVAC vector or VLP particles) immunizes first to arouse the activation of immune cells against natural trimeric Env RBD of the immune system. Of course, this part of the immune cells may account for a small part in the activated cells. Then, using the HIV RBD immunogen with mutation or glycan shield of the nonneutralizing immunodominant epitopes boosts to increase specifically the previously activated immune cells and related neutralizing antibody against RBD epitope. The engineered gp120 (called RSC3) [37, 39] which was used to obtain mAb VRC01 is a good antigen candidate of boosting. Shaping it via mutation has made it instead of binding the great majority of nonneutralizing antibodies to gp120, but bound the b12 and VRC01. In addition, we can also connect the immune-enhancing IgG Fc fragment [86, 87] to RSC3 in order to enhance the immunogenicity of RBD epitope. We hope that this new strategy can provide new ideas for AIDS vaccine research and development.

An AIDS vaccine could also be designed to induce a combination of antibodies targeting different sites, such as CD4bs and CD4i, in gp120 to confer breadth, potency, and protection [34].

## Figures and Tables

**Table 1:** Each of the listed antibodies target in different sites, including CD4bs, V1, V2, and V3 loop, membrane-proximal region of gp41 (MPER).

	Epitope	Antibody
NAbs	CD4bs	VRC01, VRC-PG04, 3BNC117/60, HJ16, 3BC176/315, NIH45-46, b12, and 1F7
	V1/V2-glycan	PG9, PG16
	V3-glycan	PGT121, PGT125, PGT135, PGT 128, 447-52D, and B48E
	Gp41	2F5, 4E10, Z13e1, and 10E8

Non-Nabs	CD4i	17b, X5, E51, 48d, and 412d
	V3	447-52D, 19b, and 14e
